# miRNA-34b as a tumor suppressor in estrogen-dependent growth of breast cancer cells

**DOI:** 10.1186/bcr3059

**Published:** 2011-11-23

**Authors:** Yee-Ming Lee, Jen-Yi Lee, Chao-Chi Ho, Qi-Sheng Hong, Sung-Liang Yu, Chii-Ruey Tzeng, Pan-Chyr Yang, Huei-Wen Chen

**Affiliations:** 1 Department and Institute of Pharmacology, College of Medicine, National Yang-Ming University, No. 155, Sec. 2, Linong Street, Taipei, 112, Taiwan; 2 Graduate Institute of Toxicology, College of Medicine, National Taiwan University, No. 1, Sec. 1, Ren-Ai Road, Taipei, 100, Taiwan; 3 Department of Internal Medicine, College of Medicine, National Taiwan University, No. 1, Sec. 1, Ren-Ai Rd, Taipei, 100, Taiwan; 4 Department of Clinical Laboratory Sciences and Medical Biotechnology, College of Medicine, National Taiwan University, No. 1, Sec. 1, Ren-Ai Road, Taipei, 100, Taiwan; 5 Department of Obstetrics and Gynecology, Taipei Medical University Hospital, No. 252, Wu Hsing Street, Taipei, 110, Taiwan

## Abstract

**Introduction:**

Estrogen is involved in several physiological and pathological processes through estrogen receptor (ER)-mediated transcriptional gene regulation. miRNAs (miRs), which are noncoding RNA genes, may respond to estrogen and serve as posttranscriptional regulators in tumorigenic progression, especially in breast cancer; however, only limited information about this possibility is available. In the present study, we identified the estrogen-regulated miR-34b and investigated its functional role in breast cancer progression.

**Methods:**

Estrogen-regulated miRNAs were identified by using a TaqMan low density array. Our *in vivo *Tet-On system orthotopic model revealed the tumor-suppressive ability of miR-34b. Luciferase reporter assays and chromatin immunoprecipitation assay demonstrated miR-34b were regulated by p53-ER interaction.

**Results:**

In this study, we identified one such estrogen downregulated miRNA, miR-34b, as an oncosuppressor that targets cyclin D1 and Jagged-1 (JAG1) in an ER+/wild-type p53 breast cancer cell line (MCF-7), as well as in ovarian and endometrial cells, but not in ER-negative or mutant p53 breast cancer cell lines (T47D, MBA-MB-361 and MDA-MB-435). There is a negative association between ERα and miR-34b expression levels in ER+ breast cancer patients. Tet-On induction of miR-34b can cause inhibition of tumor growth and cell proliferation. Also, the overexpression of miR-34b inhibited ER+ breast tumor growth in an orthotopic mammary fat pad xenograft mouse model. Further validation indicated that estrogen's inhibition of miR-34b expression was mediated by interactions between ERα and p53, not by DNA methylation regulation. The xenoestrogens diethylstilbestrol and zeranol also showed similar estrogenic effects by inhibiting miR-34b expression and by restoring the protein levels of the miR-34b targets cyclin D1 and JAG1 in MCF-7 cells.

**Conclusions:**

These findings reveal that miR-34b is an oncosuppressor miRNA requiring both ER+ and wild-type p53 phenotypes in breast cancer cells. These results improve our ability to develop new therapeutic strategies to target the complex estrogenic pathway in human breast cancer progression through miRNA regulation.

## Introduction

Breast cancer is the most frequently occurring cancer in women [1], and the majority of the cases (about 70%) are estrogen receptor (ER)-positive (ER+) [2-4]. Activated, functional ER can stimulate tumor growth and cell proliferation; therefore, it has been postulated that in most ER+ breast tumors, ER is the driving force underlying tumorigenesis, rendering it a principal target for treatment [5,6]. The agents that antagonize estrogenic action (for example, tamoxifen (TAM) and other selective estrogen receptor modulators (SERMs)) are used clinically to treat ER+ breast cancer patients. However, for some ER+ patients, these drugs are not effective for long-term use, and, in addition, many are not responsive to hormone therapy at all [7]. Therefore, the challenge is to further clarify the ER signaling pathway to identify other therapeutic targets and to develop new predictive biomarkers for better treatments.

ER signaling is complicated. ER is known to associate with numerous cofactors that act at multiple levels, including transcription, translation and even posttranslation. The classical estrogen pathway is the direct binding of estrogen-responsive elements by ligand-activated ER to regulate gene expression. Estrogen may also act as a coactivator of other transcription factors to turn on oncogenes in breast cancer in the nonclassical pathway [8-10]. Furthermore, estrogen can stimulate rapid, extranuclear (nongenomic) signaling events, such as the activation of the Src/Ras/Erk signaling pathway. Although the mechanisms of estrogen signaling in breast cancer have been extensively studied, there are still elusive interactions to be elucidated.

miRNAs (miRs) are an evolutionarily conserved class of small, noncoding RNAs of approximately 22 nucleotides that decrease gene expression posttranscriptionally by complementary binding to the mRNA 3'UTR in a sequence-specific manner, resulting in cleavage or translational repression of the target mRNA [11]. Many miRNAs have been correlated with various kinds of cancers and function as oncogenes or tumor suppressor genes [12]. Recently, the miRNA expression profile for breast cancer has been reported in a study in which comparisons between normal and tumorous breast tissues revealed that miR-10b, miR-125b and miR-145 were downregulated and that miR-21 and miR-155 were upregulated [13]. Furthermore, studies comparing miRNA profiles in breast cancer with different ER/progesterone (PR)/HER2 levels showed that specific miRNA expression levels could be correlated to different ER/PR status (miR-142-5p, miR-200a, miR-205 and miR-25) and HER2 status (let-7f, let-7g, miR-107, miR-10b, miR-126, miR-154 and miR-195) [14]. These studies suggest that miRNAs could play pivotal roles in the pathological and molecular functions in the tumorigenesis of breast cancer. Hormone-regulated miRNAs might be potential therapeutic targets or might serve as prognostic markers for hormone-dependent tumors. However, few studied have focused on hormone regulation of miRNAs in breast cancer.

To identify estrogen-regulated miRNAs in breast cancer, we examined the miRNA profile of the ER+ breast cancer cell line MCF-7 with and without estrogen treatment using a real-time, quantitative PCR (qPCR)-based TaqMan low density array (TLDA; Applied Biosystems, Foster City, CA, USA). One of the estrogen-regulated miRNAs, miR-34b, has been identified and has been functionally validated as a tumor suppressor miRNA downregulated by estrogen. We demonstrate herein that estrogen regulates the promoter activity of miR-34b gene through the interaction between ER and p53. Previous studies showed that miR-34b has important functions in cell proliferation and apoptosis and also serves as a direct transcriptional target of p53 [15-17]. Herein, on the basis of clinical and *in vivo *animal models, we show that miR-34b may play a role in controlling the growth of ER+ breast cancer. The evidence we present suggests that miR-34b is a crucial factor in ER+ breast cancer tumorigenesis and may serve as a target for miRNA-based breast cancer therapy or as a miRNA-based prognostic marker, and we provide new insights into breast cancer treatments.

## Materials and methods

### Patient tissue specimens

Specimens from 47 breast cancer patients were used for miRNA analysis. The study was approved by the Institutional Review Board of the National Taiwan University Hospital, Taipei, Taiwan. Written informed consent was obtained from all patients. Frozen sections of breast cancer tissue were obtained when patients underwent surgical resection of breast tumors at the National Taiwan University Hospital between December 2003 and December 2006. The 47 clinical samples were grouped according to pathological classification, which included the ER, PR and HER2 status. Of these specimens, 26 samples were ER+ and 21 samples were ER-negative (ER-). The clinical characteristics of the breast cancer patients are listed in Table 1. None of these patients had received adjuvant chemotherapy.

**Table 1:** Clinical characteristics of breast cancer patients

Patient demographics	ER+	ER-	*P*-value
Patients, *n *(%)	26 (55.32%)	21 (44.68%)	
Mean age ± SD (years)	53.44 ± 13.79	54.95 ± 10.13	0.68^a^
Tumor stage, *n *(%)			
Stage I	11 (42.31%)	8 (38.10%)	0.7699^b^
Stages II and III	15 (57.69%)	13 (61.90%)	
Tumor type, *n *(%)			
HER2+	15 (57.69%)	12 (57.14%)	0.9698^b^
HER2-	11 (42.31%)	9 (42.86%)	
5-year disease-free survival, *n *(%)	16 (61.54%)	13 (61.90%)	0.9795^b^

ER = estrogen receptor. ^a^*t*-test; ^b^χ^2 ^test.

### Cells and treatments

MCF-7 cells were purchased from America Type Culture Collection (ATCC; Manassas, VA, USA). The cells were maintained in 10% fetal bovine serum (FBS) as prescribed. MDA-MB-361, MDA-MB-435, T47D and OVCAR4 cells were purchased from among the NCI-60 cell lines (Developmental Therapeutics Program, National Cancer Institute, Frederick, MD, USA) and cultured in RPMI 1640 with 10% FBS as prescribed. 293T cells were purchased from ATCC and maintained in 10% FBS as prescribed. The ligands 17β-estradiol (E2), 4-hydroxytamoxifen (4-OHT), diethylstilbestrol (DES) and zeranol (ZEA) were purchased from Sigma-Aldrich (St Louis, MO, USA). Before MCF-7 cells were treated with ligands, the medium was replaced with phenol red free α-MEM (Life Technologies, Rockville, MD, USA) containing 10% dextran charcoal-stripped FBS (Thermo Fisher Scientific Inc, Waltham, MA, USA) for 3 days. Cells were treated with 10 nM E2, 100 nM 4-OHT, 10 nM DES or 10 nM ZEA for 24 hours prior to analyses.

### Proliferation assay

A 3-(4,5-dimethylthiozol-2-yl)-2,5-diphenyltetrazolium bromide (MTT) (Sigma-Aldrich) assay was performed to determine cell proliferation. Briefly, MCF-7 cells were plated in 96-well plates at a density of 5 × 10^3^/well. After 24-hour incubation, cells were serum-starved overnight. The cells were then treated with different concentrations of estrogen for indicated times, then culture medium containing 0.5 mg/ml MTT solution was added to each well. After 1.5-hour incubation, the medium was removed and dimethyl sulfoxide was added to the wells. The color intensity of solubilized formazan was measured at 570 nm with an ELISA plate reader (VICTOR *X*3 Multilabel Plate Reader; PerkinElmer, Waltham, MA, USA).

### Anchorage-dependent colony formation assay

Six-well plates were used for seeding 100 cells in α-MEM supplemented with 10% FCS. E2 and 4-OHT were added to the plate at different concentrations every 2 days. Plates were incubated in a 37°C incubator for 2 weeks. The number of colonies was counted after they were stained with 0.05% crystal violet (Sigma-Aldrich) for 1 hour and washed extensively with PBS.

### TaqMan Low Density Array

The human miRNA TLDA contains eight sample-loading lines, each of which is connected by a microchannel to 48 miniature reaction chambers for a total of 384 wells per card. It contains 365 different human miRNA assays plus two carefully selected small nucleolar RNAs (snRNAs) that function as endogenous controls for data normalization. The human miRNA TLDA was used in conjunction with Multiplex reverse transcriptase (RT; Applied Biosystems) consisting of eight predefined RT primer pools containing up to 48 RT primers each. All 365 miRNA targets were reverse-transcribed in eight separate RT reactions, and each RT reaction was pipetted into one of the eight filling ports on the TaqMan array. To perform PCRs, we used a 7900HT Fast Real-Time PCR System (Applied Biosystems), and SDS version 2.3 software (Applied Biosystems) was used for comparative differential expression (ΔCt) analysis.

### Quantitative real-time PCR analysis of miRNA expression

Total RNAs were extracted from cells using TRIzol reagent (Invitrogen/Life Technologies, Carlsbad, CA, USA). miRNAs were quantified using TaqMan miRNA assays (Applied Biosystems). U6B was used for normalization of miRNA expression. The PCR was run in a 7900HT Fast Real-Time PCR System, and SDS version 2.3 software (Applied Biosystems) was used for comparative ΔCt analysis. Experiments were performed three times in triplicate.

### Western blot analysis

MCF-7 cells were treated with the chemicals described above. Cells were washed twice with ice-cold PBS and collected for protein extraction. Protein was extracted by using mammalian protein extraction reagent (Pierce Biotechnology, Inc, Rockford, IL, USA) containing protease inhibitors (Sigma-Aldrich). SDS-PAGE with 10% resolving gel was used to separate proteins (25 mg/lane). Mouse anti-cyclin D1 mAb (Merck KGaA, Darmstadt, Germany), mouse anti-Jagged-1 (anti-JAG1) mAb (Santa Cruz Biotechnology, Santa Cruz, CA, USA) and mouse anti-β-actin mAb (Santa Cruz Biotechnology) were all used according to the manufacturer's instructions. Bound antibodies were detected using an enhanced chemiluminescence system (Santa Cruz Biotechnology). Chemiluminescent signals were captured using the Fujifilm LAS-3000 system (Fujifilm, Tokyo, Japan). All experiments were performed at least three times in triplicate.

### Vectors and constructs

We used the p*Silencer *4.1-CMV puro vector (Applied Biosystems), the pTRE-Tight vector (631059; Clontech Laboratories, Inc, Mountain View, CA, USA) and the Tet-On vector (Clontech Laboratories, Inc) in the plasmid constructs. A precursor form of miR-34b was inserted into the p*Silencer *4.1-CMV puro vector, then subcloned into the pTRE-Tight vector for overexpression. Primer sequences are shown in Additional file 1, Supplementary Table 1.

### Stable clone and precursor miRNA transfection

MCF-7 cells were plated in six-well plates at a density of 5 × 10^5 ^cells per well in Opti-MEM Reduced Serum Medium (Life Technologies) supplemented with 10% FBS. Lipofectamine 2000 transfection reagent (Invitrogen/Life Technologies) and pTRE-miR-34b and Tet-On plasmids were mixed according to the manufacturer's instructions and added to the cells. After 6 hours of incubation, the medium was changed with fresh α-MEM plus 10% FBS. After cotransfection of the two plasmids, stable clones were selected by addition of neomycin (1 mg/μl). Precursor miRNAs, antagomirs and negative controls (mock transfection) were obtained from Applied Biosystems. For precursor miRNA, cells were transfected with Lipofectamine LTX (Invitrogen/Life Technologies) with 6 nM precursor miRNA and harvested 48 or 72 hours later.

### Methylation-specific PCR and bisulfate sequencing

Genomic DNA was extracted from MCF-7 cells using the QIAamp DNA Mini Kit (QIAGEN, Valencia, CA, USA). Genomic DNA (2 μg) was modified by sodium bisulfite using the EZ DNA Methylation Kit (Zymed Research Corp, Irvine, CA, USA). Methylation-specific PCR (MSP) and bisulfite-sequencing PCR (BSP) analysis were then performed as described previously [18]. Primers for MSP and BSP were designed by using the MethPrimer software program [19]. Primer sequences are shown in Additional file 1, Supplementary Table 1.

### Promoter reporter assay

Upstream regions of miR-34b/c were amplified by PCR and then cloned into the pGEM-T Easy Vector System (Promega, Madison, WI, USA). Each PCR primer carried a 5' overhang that contained a *Hin*dIII recognition site. After verification of the sequences, miR-34b/c fragments were excised using *Hin*dIII and ligated into a pGL3-Basic vector (Promega). In addition, oligonucleotide fragments corresponding to p53-responsive elements were synthesized and inserted upstream of the miR-34b/c promoter in the pGL3-Basic vector. Cells (5 × 10^4 ^per well in 24-well plates) were transfected with 100 ng of one of the reporter plasmids using Lipofectamine 2000 reagent (Invitrogen/Life Technologies). For cotransfection of reporter genes with ER expression plasmid, equal numbers of cells were cotransfected with 100 ng of one of the reporter plasmids, 100 ng of pcDNA 3.1-ER (Invitrogen/Life Technologies) or an empty vector. The pGL3-Basic vector without an insert served as the negative control. Luciferase activity was measured 48 hours after transfection using a Dual-Luciferase Reporter Assay System (Promega). Primer sequences are shown in Additional file 1, Supplementary Table 1.

### Luciferase reporter assay

The 3'UTRs of human JAG1 and cyclin D1 were amplified from human genomic DNA and cloned into a pMIR-REPORT miRNA Expression Reporter Vector System (Ambion/Life Technologies) by directional cloning. The resultant plasmid was designated Luc-JAG1-3'UTR-WT and Luc-CyclinD1-3'UTR-WT. To generate the Luc-JAG1-3'UTR-Mut and Luc-CyclinD1-3'UTR-Mut construct, seed regions were mutated by removing complementary nucleotides of miR-34b. 293T cells (3 × 10^5 ^per well) were cotransfected with a 0.5-μg firefly luciferase reporter vector and a 0.05-μg control vector containing *Renilla *luciferase, pRL-SV40 (Promega), using Lipofectamine 2000 reagent (Invitrogen/Life Technologies) in six-well Costar plates (Corning Life Sciences, Lowell, MA, USA). For each well, 200 pM miR-34b precursor molecule (Ambion/Life Technologies) or a negative control (mock) precursor miRNA (Ambion/Life Technologies) was cotransfected with the reporter constructs. Luciferase assays were performed 24 hours after transfection (Dual-Luciferase Reporter Assay System; Promega). Firefly luciferase activity was normalized to *Renilla *luciferase activity.

### Chromatin immunoprecipitation assay

The chromatin immunoprecipitation (ChIP) assays were carried out as described previously [20]. Chromatin was immunoprecipitated for 16 hours at 4°C using 10 μl each of anti-RNA polymerase II, clone CTD4H8 (Millipore, Billerica, MA, USA), normal mouse immunoglobulin G (IgG; Millipore) and anti-ERα, clone HC-20 (Santa Cruz Biotechnology). In addition, 1/100 of the solution collected before adding the antibody was used as an internal control for the amount of input DNA. PCR was carried out in a 20-μl volume containing 1/100 of the immunoprecipitated DNA, blend Taq polymerase and 5 μM of each primer. The PCR protocol entailed initial denaturation for 3 minutes at 94°C and 33 cycles of 20 seconds at 94°C, 30 seconds at 59°C and 30 seconds at 72°C, followed by a final extension at 72°C for 2 minutes. Primer sequences are shown in Additional file 1, Supplementary Table 1.

### Orthotopic mammary fat pad xenograft mouse model

This study was approved by the Institutional Review Board and the Animal Care and Use Committee at National Taiwan University. The survival rate of transfected MCF-7 cells was determined by counting cell numbers using trypan blue. Transfected MCF-7 cells (1 × 10^6 ^live cells in 100 μl Hank's balanced salt solution) were injected into the mammary fat pads of 5-week-old NOD SCID mice (supplied by the animal center at the College of Medicine, National Taiwan University). To determine whether miR-34b could reduce orthotopic tumor growth, the mice were randomized into two groups (*n *= 10 for each group): (1) miR-34b Tet-On overexpression stable clone without doxycycline (Sigma-Aldrich) or (2) miR-34b Tet-On overexpression clone with doxycycline. Mice were injected with doxycycline (2 mg/ml) in water [21,22] after tumor size reached 5 mm^3^. Tumor sizes were monitored every 7 days by using electronic vernier calipers, and the tumor volume (*V*) was calculated by using the formula *V *= 0.4 × *ab*^2^, where *a *and *b *are the longest and shortest diameters of each tumor, respectively.

### Immunohistochemistry

We performed immunohistochemistry by using 5-μm formalin-fixed, paraffin-embedded tissue sections, placing them on silane-coated slides and baking them at 70°C overnight. Afterward the slides were deparaffinized, hydrated, placed in 10 mM citrate buffer (pH 6) and microwaved for a total of 20 minutes for antigen retrieval. After cooling, the slides were treated with 3% H_2_O_2 _for 15 minutes. Appropriate blocking (3% BSA and 0.2% Triton X-100) for 1 hour and anti-cyclin D1, clone H-295 (Santa Cruz Biotechnology) rabbit pAb was used. Rabbit IgG (Millipore) was used as a negative control. We used the VECTASTAIN Elite ABC Kit (Vector Laboratories, Burlingame, CA, USA) for detection. Chromagen diaminobenzidine (Vector Laboratories) was used, and sections were counterstained with H & E.

### Statistical analysis

All experiments were performed in triplicate and analyzed for significant differences by analysis of variance using Microsoft Excel software (Microsoft Corp, Redmond, WA, USA). *P *< 0.05 was considered statistically significant. Where appropriate, the data are presented as means ± SD.

## Results

### Estrogen-regulated miRNA profile in ER+ breast cancer cells

To investigate whether estrogen regulates miRNA expression, the ER+ human breast cancer cell line MCF-7 was treated with 10 nM E2 for 24 hours, and changes in the miRNA expression profile were analyzed by performing qPCR-based TLDA miRNA assays. Table 2 lists the 26 miRNAs that were significantly downregulated and the 28 miRNAs that were significantly upregulated (greater than fivefold changes) by E2 treatment of MCF-7 cells. Among these estrogen-regulated miRNAs, miR-34b has been shown to regulate tumor progression in a variety of cancers [15-17,23-25]. Because E2 markedly decreased miR-34b expression levels (72-fold), miR-34b was chosen for further study.

**Table 2:** Estrogen-regulated miRNA profiles of ER+ breast cancer cell line MCF-7 using TaqMan Low-Density Array

Downregulated miRNAs			Upregulated miRNAs		
**miRNAs**	**Fold change**	***P*-value**	**miRNAs**	**Fold change**	***P*-value**

miR-509	-119.389	<0.001	miR-189	5.024565	0.002
miR-376a	-102.459	<0.001	miR-326	5.52856	<0.001
miR-629	-91.9202	<0.001	miR-520c	5.619023	<0.001
miR-34b	-71.9166	<0.001	miR-520b	5.787208	<0.001
miR-519d	-63.3272	<0.001	miR-329	6.293726	<0.001
miR-33	-41.4439	<0.001	miR-17-3p	7.373741	<0.001
miR-515-5p	-40.7382	<0.001	miR-369-3p	8.591994	<0.001
miR-624	-38.3289	<0.001	miR-519c	8.613923	<0.001
miR-34c	-37.07	<0.001	miR-524	11.21323	<0.001
miR-551b	-33.1565	<0.001	miR-576	13.31873	<0.001
miR-564	-27.6564	<0.001	miR-653	13.9337	<0.001
miR-449	-25.5663	<0.001	miR-383	15.40733	0.004
miR-10b	-22.5561	<0.001	miR-627	15.45773	<0.001
miR-518e	-22.0561	<0.001	miR-105	16.18497	0.001
miR-139	-21.8713	<0.001	miR-616	20.96113	0.032
miR-520h	-18.821	<0.001	miR-422b	24.21192	0.018
miR-453	-17.2921	<0.001	miR-452	28.38138	<0.001
miR-526b	-10.5159	0.014	miR-380-5p	33.71417	0.007
miR-371	-9.59159	<0.001	miR-548b	42.83806	<0.001
miR-656	-8.11175	0.001	miR-521	44.27376	0.005
miR-124a	-6.73618	<0.001	miR-579	45.54916	<0.001
miR-518f	-5.80659	0.002	miR-142-5p	51.74748	<0.001
miR-135a	-5.40944	<0.001	miR-519e	53.65128	<0.001
miR-485-5p	-5.01188	0.003	miR-185	55.94657	<0.001
			miR-493	92.40058	<0.001
			miR-515-3p	97.85771	<0.001

ER = estrogen receptor. The expression profiles of miRNAs which have at least fivefold changes in expression. We used U6B expression as the internal control to normalize expression levels between the two groups. Experiments were repeated in triplicates. Relative expression = 2^^-(with 10 nM estradiol/without estradiol)^.

### Negative correlation of miR-34b expression level and ER status in ER+ human breast cancers

**Figure 1:**
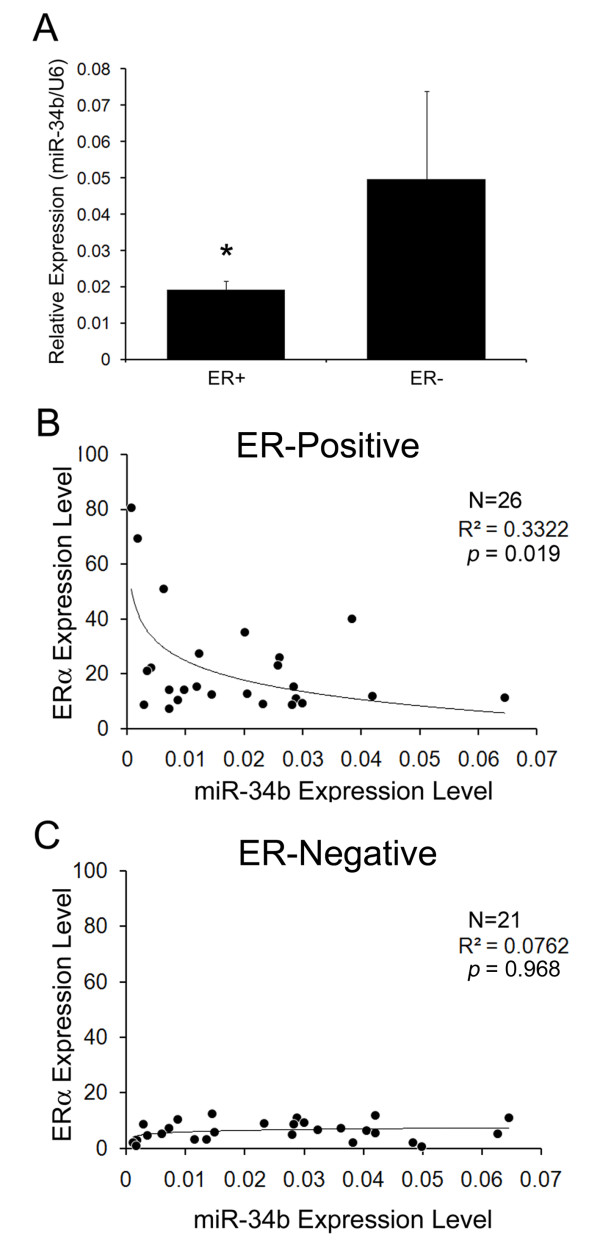
**Negative correlation of miRNA (miR)-34b expression level and estrogen receptor (ER) status in ER+ human breast cancers**. **(A) **miR-34b expression in human breast cancer tissue. miR-34b expression levels were analyzed by quantitative PCR (qPCR) (ER+, *n *= 26; ER-, *n *= 21). The results are normalized to internal U6 snRNA expression. **(B) **Correlation analysis of ERα and miR-34b in ER+ human breast cancer tissues. *P *= 0.019. **(C) **Correlation analysis of ERα and miR-34b in ER- human breast cancer tissues. *P *= 0.968. For both parts **(B) **and **(C)**, ERα mRNA expression levels were analyzed performing SYBR Green qPCR for each tissue sample. Data represent means ± SD of triplicate experiments (*P < 0.05). The results are normalized to internal TaqMan Low-Density Array expression.

We next examined the expression levels of miR-34b in 47 human breast cancer patients (Table 2). We found that the ER+ tissues had lower miR-34b expression levels compared to ER- tissues (ER+: *n *= 26 vs ER-: *n *= 21; *P *< 0.05) (Figure 1A). Moreover, the correlation between miR-34b and ER expression levels were inversely correlated (Pearson coefficient = -0.4185, *P *= 0.0019) for ER+ patients (Figure 1B). However, the correlation of miR-34b and ER showed no significance for ER- patients (Pearson coefficient = 0.009, *P *= 0.9679) (Figure 1C). These data suggest that miR-34b expression is closely linked to ERα expression levels in ER+ human breast tumors involved in the complexity of *in vivo *systems.

### Estradiol increased anchorage-dependent colony formation and repressed miR-34b expression in MCF-7 cells

**Figure 2:**
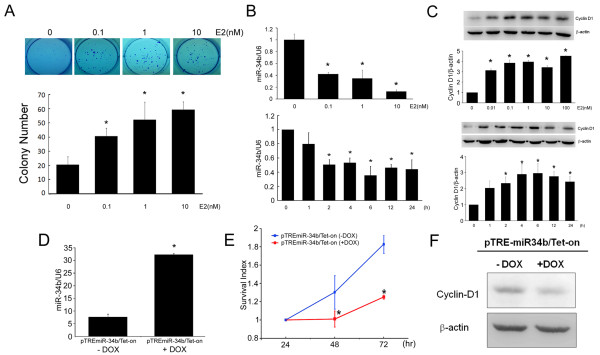
**Estradiol (E2) increased anchorage-dependent colony formation and repressed miRNA (miR)-34b expression**. **(A) **Anchorage-dependent colony formation assay results. MCF-7 cells were seeded into six-well plates and treated with different concentrations of E2 (0 to 10 nM) for 14 days. Colonies were fixed and stained with crystal violet dye. Representative results are shown in the upper panel. The number of colonies are shown in the bottom panel (*n *= 3). **(B) **miR-34b expression of MCF-7 cells treated with E2 (10 nM) for different time periods (*n *= 3) or with different concentrations of E2 for 24 hours (*n *= 3). The results were normalized to internal U6 snRNA expression. **(C) **Western blots of cyclin D1 in MCF-7 cells treated with E2 (10 nM) for different time periods (*n *= 3) or with different concentrations of E2 for 24 hours (*n *= 3). β-actin was probed as an internal control. The results are expressed as means ± SD. *P < 0.05. **(D) **Stable clones of pTRE-miR-34b/Tet-On were induced by doxycycline (DOX) (2 μM) for 48 hours, and miR-34b expression was analyzed by performing quantitative PCR. **(E) **Cell viability of pTRE-miR-34b/Tet-On in the presence or absence of DOX (2 μg/ml) was detected by using 3-(4,5-dimethylthiozol-2-yl)-2,5-diphenyltetrazolium bromide (*n *= 3). **(F) **Western blot showing cyclin D1 after miR-34b induction. β-actin was probed as an internal control.

**Figure 3:**
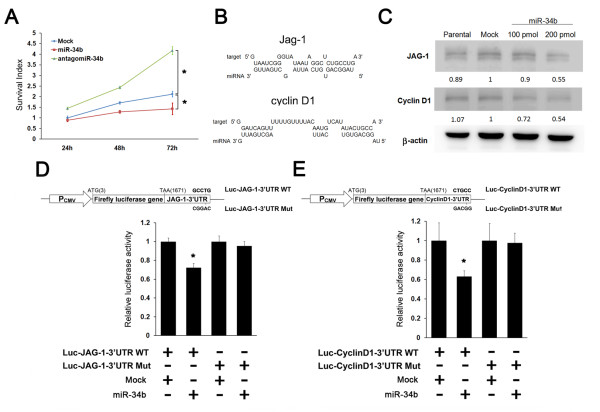
**miRNA (miR)-34b suppressed cell proliferation by directly regulating JAG1 and cyclin D1**. **(A) **Cell viability after transfection of negative control (Mock) (200 pM), precursor miR-34b (200 pM) or antagomir-34b (200 pM) was detected by using 3-(4,5-dimethylthiozol-2-yl)-2,5-diphenyltetrazolium bromide (*n *= 3). **(B) **Predicted binding structures of miR-34b with targets JAG1 and cyclin D1. RNAhybrid software was used for nucleotide-binding sequence prediction. **(C) **Western blots showing JAG1 and cyclin D1 after 24-hour treatment with precursor miR-34b or (200 pM) in MCF-7 cells. β-actin was probed as internal control. The numbers under the blots represent the semiquantitative values of the Western blot analysis. **(D) **and **(E) **Top: Diagrams depicting the pMIR-REPORT vector luciferase reporter constructs containing a cytomegalovirus (CMV) promoter (P_CMV_), which was used to verify the putative miR-34b binding sites. Bottom bar graphs: 293T cells were cotransfected with negative control (Mock) (200 pM) or miR-34b (200 pM), Luc-JAG1-3'UTR (0.5 μg) or Luc-CyclinD1-3'UTR (0.5 μg), along with a pRL-SV40 reporter plasmid (0.05 μg). After 24 hours, luciferase activity was measured. Values are presented as relative luciferase activity after normalization to *Renilla *luciferase activity. Data are expressed as means ± SD of results derived from triplicate experiments. *P < 0.05.

To verify that miR-34b expression is linked to tumor growth, MCF-7 cells were treated with different concentrations of E2 (0.1, 1.0, 10 and 100 nM), and cell proliferation rates were determined. E2 increased anchorage-dependent growth in a concentration-dependent manner (Figure 2A), but the expression levels of miR-34b decreased concentration-dependently. E2 (10 nM) treatment reduced miR-34b expression by 88% in MCF-7 cells (Figure 2B). The inhibitory effect of estrogen on miR-34b expression was apparent within 2 hours and was sustained for at least 24 hours in MCF-7 cells (Figure 2B). Transfection of precursor miR-34b or antagomir-34b also showed significant decreases or increases in cell proliferation (Figure 3A). These data suggest that miR-34b may serve as a tumor suppressor that regulates tumor progression, as has been reported in a variety of cancers [15-17,23-25].

### miR-34b suppressed cell proliferation by direct regulation of Jagged-1 and cyclin D1

Previous studies have shown that high expression of cyclin D1 and Jagged-1 correlate with poor prognosis in breast cancer [26-28]. According to miRNA target gene prediction software, including PicTar, miRanda and miRNAMap, miR-34 could target key regulators of cell proliferation, including cyclin D1 and JAG1 (Figure 3B). To investigate the possible link between miR-34b and these possible downstream targets, the protein expression levels of cyclin D1 and JAG1 were examined. As shown in Figure 2C, both cyclin D1 and JAG1 were upregulated by treatment with E2 in a concentration- and time-dependent manner. Moreover, overexpression of precursor miR-34b leads to downregulation of JAG1 and cyclin D1 (Figure 3C).

To further confirm the interaction between miR-34b and its putative target genes, we cloned the JAG1 and cyclin D1 3'UTR sequence and inserted it downstream of the firefly luciferase coding region of the pMIR-REPORT vector (Figures 3D and 3E). Mutants with the putative binding sites were prepared as described above (see Materials and methods). Our results show that the luciferase activity of the wild-type JAG1 and cyclin D1 3'UTR construct was significantly inhibited after the introduction of miR-34b into 293T cells, but not by the negative control. Mutations of the 3'UTR-binding sites completely abolished the ability of miR-34b to regulate luciferase expression (Figures 3D and 3E). These results demonstrate that JAG1 and cyclin D1 are potential targets of miR-34b. Furthermore, these data suggest that miR-34b may suppress tumor growth through the suppression of cyclin D1 and JAG1 expression.

### miR-34b inhibited tumor growth in mouse mammary fat pads xenografted with MCF-7 pTRE-miR-34b/Tet-On stable clones

**Figure 4:**
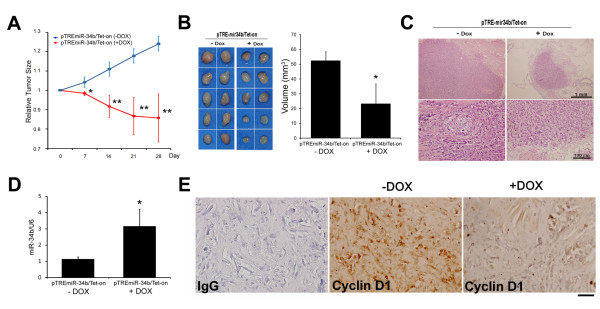
**miRNA (miR)-34b inhibited tumor growth in mouse mammary fat pads xenografted with MCF-7 pTRE-miR-34b/Tet-On stable clones**. **(A) **Tumor size was calculated by using the formula *V *= 0.4 × *ab*^2^, where *a *and *b *are the longest and shortest diameters of each tumor, respectively. Relative tumor size was normalized by the tumor size measured at day 0 (*n *= 10). Day 7, *P *= 0.05; day 14, *P *= 0.0079; day 21, *P *= 0.0052; day 28, *P *= 0.0061. **(B) **Left: Photomicrographs of orthotopic tumors excised from mice. Right: Tumor volume of the orthotopic tumor tissues from doxycycline (DOX)-treated mice (*n *= 10). **(C) **H & E-stained tumor tissue sections from each group. **(D) **Quantitative analysis of miR-34b expression in orthotopic tumors (*n *= 10). The results are normalized to internal U6 snRNA expression and represent means ± SD. *P < 0.05; **P < 0.01. **(E) **Immunohistochemical staining of proliferation marker cyclin D1 in orthotopic tumor tissues. IgG = immunoglobulin G. Formalin-fixed, paraffin-embedded tissue sections were prepared and stained as indicated. Bar = 200 μM.

Since we observed that miR-34b was downregulated in ER+ breast cancer cells (MCF-7 cells), we speculated that overexpression of miR-34b might inhibit cell proliferation. To test this hypothesis, we established a miR-34b Tet-On overexpression system. In this system, miR-34b is conditionally turned on by doxycycline treatment (Figure 2D). Addition of doxycycline significantly decreased cell proliferation (Figure 2E) and cyclin D1 expression (Figure 2F) in MCF-7/miR-34b Tet-On stable clones. Doxycycline did not exert significant effects on cell growth and cyclin D expression in parental and mock transfection of MCF-7 cells (Additional file 2, Figure S1). To test whether miR-34b suppresses tumor growth *in vivo*, 1 × 10^6 ^MCF-7/miR-34b Tet-On stable clones were injected into the mammary fat pads of female NOD SCID mice. Tumor size increased significantly with time. Doxycycline was applied to induce miR-34b expression after tumor size reached 5 mm^3^. Seven days after induction, the induced miR-34b group showed significant reduction in tumor size compared to the control group (*P *< 0.05) (Figure 4A). After 28 days, the mice were killed and the tumor volumes were compared. As shown in Figure 4B, the tumor volumes in the induced miR-34b group were significantly lower than in the control group (*P *< 0.01). H & E staining of the fat pads confirmed that miR-34b induction led to a decrease in tumor size (Figure 4C). The miR-34b expression level in Tet-On group was increased 2.76-fold compared to the noninduced miR-34b group (Figure 4D). In agreement with these findings, the expression levels of cyclin D1 were significantly reduced in the doxycycline induced group as shown by immunohistochemical staining (Figure 4E).

### p53-dependent regulation of miR-34b expression by estradiol

To investigate how E2 regulates miR-34b, we screened the miR-34b promoter for the presence of ER-responsive elements. However, we could not find any possible binding sites of ER in the miR-34b promoter. This result showed that E2 regulation of miR-34b does not occur through classical ER signaling. Recent studies showed that p53 could bind to miR-34b/c and regulate its expression [16]. Furthermore, the interaction between p53 and ER at the promoter site plays a pivotal role in the regulation of miR-34b expression [29-31].

**Figure 5:**
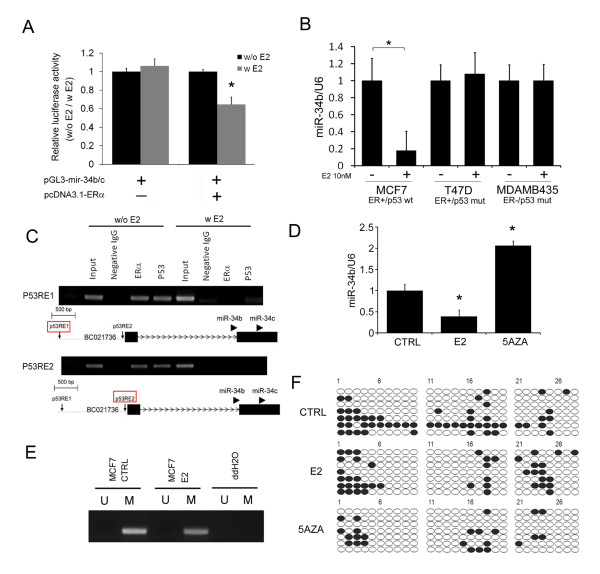
**p53-dependent regulation of miRNA (miR)-34b expression by 17β-estradiol (E2)**. **(A) **Reporter plasmids in which the luciferase coding sequence that had fused to the miR-34b promoter region were transfected into 293T cells in conjunction with either pcDNA3.1 plasmid coding with ER were cotransfected. Normalized luciferase activities are presented (*n *= 4). **(B) **miR-34b expression after E2 treatment in a subset of breast cancer cells (MCF-7, T47D and MDA-MB-435) with different estrogen receptor (ER) and p53 status (*n *= 3). The results are normalized to internal U6 snRNA expression and represent means ± SD. *P < 0.05. wt = wild type; mut = mutation. **(C) **Chromatin immunoprecipitation (ChIP) assays of MCF-7 cells treated with or without 10 nM E2. The results of PCRs of the two p53-responsive elements after ChIP with ERα or p53 in MCF-7 cells are indicated. Input = 1% of total lysate. IgG = immunoglobulin G. **(D) **Quantitative PCR of miR-34b expression in MCF-7 cells following 10 nM E2 treatment (24 hours) and 2 μM 5-aza-2'-deoxycytidine (5-AZA) (72 hours) (*n *= 3). The results are normalized to internal U6 snRNA expression and represent means ± SD. *P < 0.05. CTRL = control. **(E) **Methylation-specific PCRs of miR-34b/c CpG islands in MCF-7 cells after 10 nM E2 treatment. Bands in the ''M'' lanes are PCR products obtained with methylation-specific primers. Those in the ''U'' lanes are products obtained with non-methylation-specific primers. **(F) **Bisulfite sequencing of miR-34b/c CpG islands in the indicated breast cancer cell lines with the same treatments shown in part **(D)**. Open and filled circles represent unmethylated and methylated CpG sites, respectively. Each treatment group was assayed in seven replicate experiments.

We first demonstrated that miR-34b promoter activity was significantly inhibited by 10 nM E2 treatment of 293T cells cotransfected with ER (Figure 5A). In additional experiments, we used breast cancer cell lines bearing different p53 and ER status (MCF-7, T47D and MDA-MB-435). Only cells expressing both wild-type p53 and ERα responded to estrogen repression of miR-34b (Figure 5B). These data suggest that both ER and p53 activity are required for E2 to repress miR-34b. We next examined whether both proteins bind to the two p53 binding sites in the miR-34b promoter. ChIP assays confirmed that both p53 and ER bind to the miR-34b/c promoter in the absence of E2 (Figure 5C). Binding of p53 to the two p53RE binding sites of the miR-34b promoter region were disrupted by treatment of cells with E2 (Figure 5C). These data support the notion that E2's binding to ER may affect p53's binding to miR-34b promoter through p53-ER interaction.

Previously presented evidence indicated that miR-34b/c expression may be regulated by DNA methylation [18]. To investigate whether E2 could regulate miR-34b/c through DNA methylation, we examined the methylation status of the miR-34b/c promoter. Treatment of MCF-7 cells with a DNA methyltransferase inhibitor, 5-aza-2'-deoxycytidine (5-aza-dC), increased the expression level of miR-34b (Figure 5D) and reduced the methylation status in the CpG islands of the miR-34b promoter region as demonstrated by MSP and BSP (Figure 5E). However, treatment of cells with E2 did not cause significant differences in DNA methylation (Figure 5F). These data suggest that E2 did not regulate miR-34b/c expression through DNA methylation in MCF-7 cells.

### Xenoestrogens and tamoxifen regulate miR-34b expression in breast cancer cells

**Figure 6:**
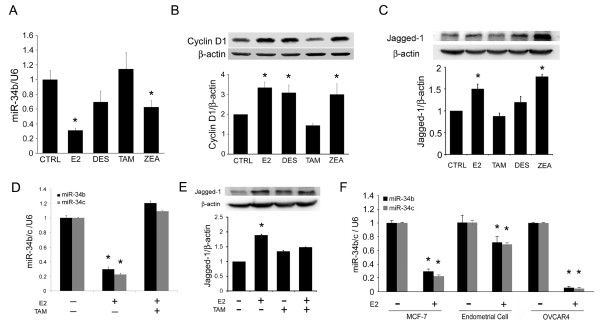
**Xenoestrogen- and tamoxifen-regulated miRNA (miR)-34b expression in breast cancer cells**. **(A) **miR-34b expression in MCF-7 cells after 24-hour treatment with estradiol, xenoestrogens or tamoxifen (*n *= 3). The results are normalized to internal U6 snRNA expression. CTRL = control; E2 = 10 nM 17β-estradiol; DES = 100 nM diethylstilbestrol; ZEA = 100 nM zeranol; TAM = 100 nM tamoxifen. **(B) **Western blot of cyclin D1 after 24-hour treatment with E2, xenoestrogens or TAM in MCF-7 cells. β-actin was probed as an internal control. Graphs at bottom present the Western blot analysis quantitative data (*n *= 3). **(C) **Western blot of JAG1 in MCF-7 after 24-hour treatment with E2, xenoestrogens or tamoxifen (*n *= 3). E2 = 10 nM estradiol; DES = 100 nM diethylstilbestrol; ZEA = 100 nM zeranol; TAM = 100 nM tamoxifen. β-actin was probed as an internal control. **(D) **Quantitative PCR of miR-34b/c expression in MCF-7 cells following 10 nM E2, 100 nM TAM or combined treatment (n = 3). The results were normalized to internal U6 snRNA expression. **(E) **Western blot showing JAG1 in MCF-7 after 24-hour treatment with E2, tamoxifen or combined treatment (*n *= 3). E2 = 10 nM estradiol; TAM = 100 nM tamoxifen. β-actin was probed as an internal control. **(F) **Quantitative expression of miR-34b and miR-34c after E2 treatment in MCF-7 breast cancer cells, mouse primary endometrial cells and OVCAR4 ovarian cancer cells (*n *= 3). The results were normalized to internal U6 snRNA expression and are expressed as means ± SD. *P < 0.05.

Some xenoestrogens may play a causative role in breast cancer tumorigenesis [32-35]. To investigate whether xenoestrogen could promote cancer progression by regulating miR-34b (similarly to E2), we examined miR-34b and its downstream target's expression level after xenoestrogen treatment. Our results show that the xenoestrogens DES and ZEA downregulate miR-34b expression in MCF-7 cells (Figure 6A). TAM, a SERM that exhibits antiestrogenic effects in breast tissue, did not affect miR-34b expression levels in MCF-7 cells (Figure 6A). In agreement with this finding, the protein levels of cyclin D1 and JAG1 were upregulated by treatment with DES and ZEA, whereas TAM had no significant effect (Figures 6B and 6C). Importantly, combined estrogen and TAM treatment reversed the effect of estrogen on miR-34b (Figures 6D and 6E).

To investigate whether the regulation of miR-34b expression is tissue-specific, we studied human OVCAR4 ovarian cancer cells and primary culture of mouse endometrial cells. Both of these cells are p53+/+ and E2-responsive. As shown in Figure 6F, E2 downregulated miR-34b/c expression in both OVCAR4 and endometrial cells. These data suggest that the E2 regulation of miR-34b/c maybe a universal event and might also contribute to the proliferation potential in these tissues.

### miR-34b/c suppresses cell growth in breast cancer cell lines

**Figure 7:**
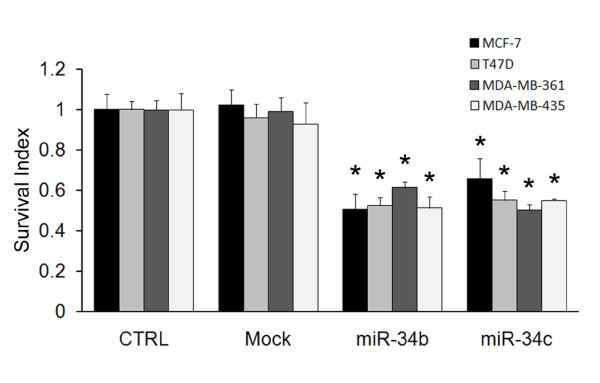
**miRNA (miR)-34b/c suppresses cell growth in breast cancer cell lines**. Cell proliferation assay of breast cancer cell lines after overexpression miR-34b/c (*n *= 6). A 3-(4,5-dimethylthiozol-2-yl)-2,5-diphenyltetrazolium bromide assay was used to monitor viable cell numbers 48 hours after transfection of miR-34b/c precursor cells. CTRL = control. The results represent means ± SD. *P < 0.05.

To evaluate this possibility by using miR-34b/c as potential therapeutic targets, we obtained four breast cancer cell lines with different ER and p53 status and overexpressed miR-34b/c. Among the four cell lines, MCF-7 expresses ER+ and wild-type p53 (ER+/p53^wt^), both T47D and MDA-MB-361 are ER+ and p53-mutant (ER+/p53^mut^), and MDA-MB-435 is ER- and p53-mutant (ER-/p53^mut^). As shown in Figure 7, despite the different status of ER and p53, all four cell lines showed a significant decrease in growth after 48 hours with the expression of miR-34b/c. This result demonstrates that the induction of miR-34b/c could suppress cell growth and that the effect was not influenced by ER or p53 status.

## Discussion

**Figure 8:**
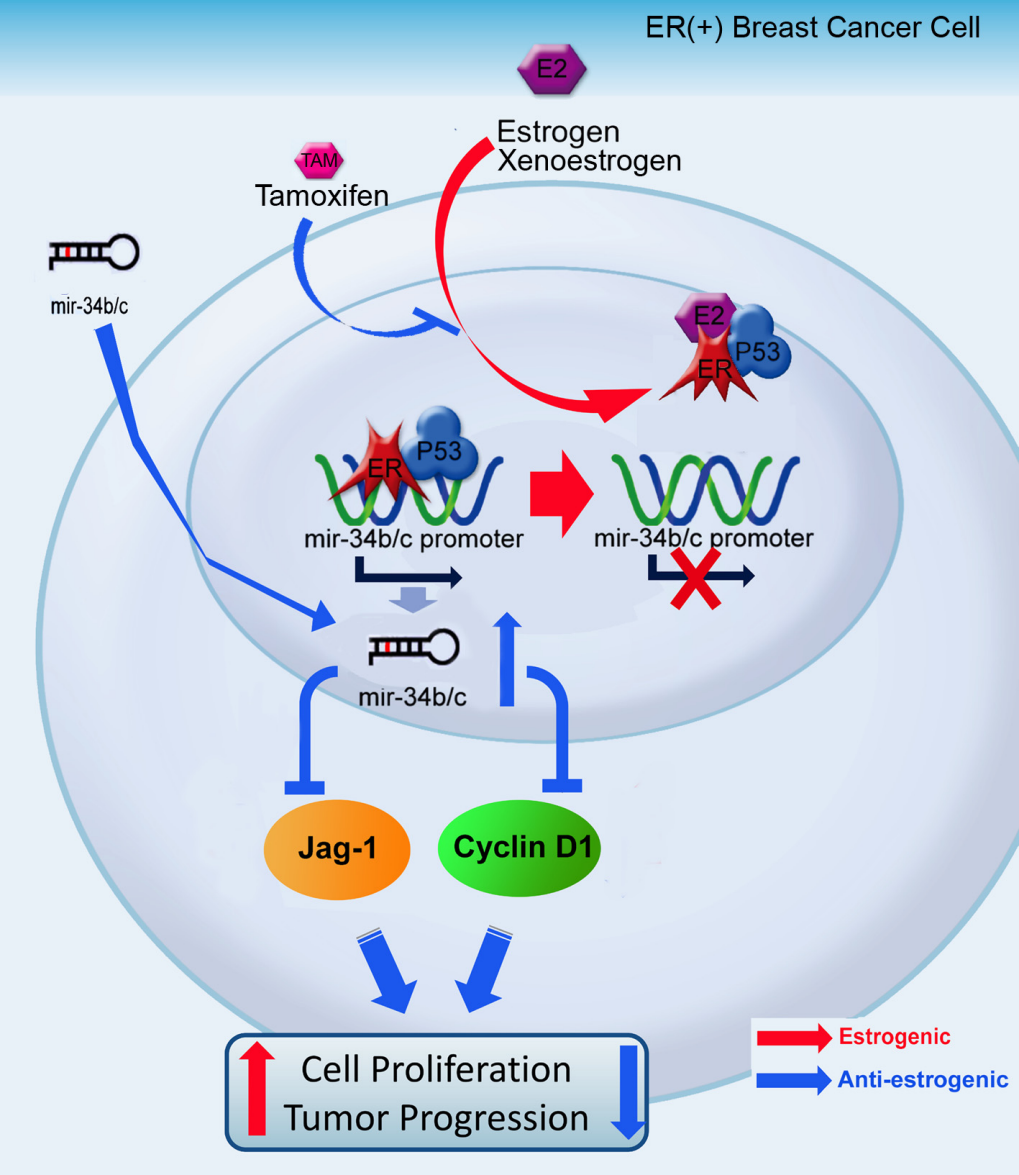
**Schematic illustrating estrogen-dependent regulation of miRNA (miR)-34b expression, its target genes and tumor growth**. The role of estrogen in the miR-34b-regulating pathway is shown. Under 17β-estradiol (E2)-free conditions, p53 and estrogen receptor (ER) bind to the miR-34b promoter and activate its transcription. However, in the presence of E2 or xenoestrogens, the activated ER disrupts the binding of the p53-ER complex to the miR-34b promoter region, which subsequently inhibits the tumor-suppressing network and promotes cell proliferation and tumor progression.

Hormone-regulated miRNAs may be potential therapeutic targets in hormone-dependent tumors. However, few studies have focused on hormone-regulated miRNA expression in breast cancer. In the present study, we analyzed the estrogen-regulated miRNA expression profile in MCF-7 breast cancer cells. We found a set of 54 miRNAs that could be regulated by E2. Among these estrogen-regulated miRNAs, miR-34b was selected for further investigation because it has been reported to be a tumor suppressor in colorectal cancer [18], prostate cancer [25] and gastric cancer [24]. In this study, we have demonstrated for the first time that miR-34b significantly regulates the growth of ER+ breast cancer cells *in vitro*. We provide evidence that miR-34b plays a pivotal role in the human breast cancer-expressing ER+ phenotype (Figure 8). By establishing Tet-On-inducible miR-34b expression in MCF-7 cells, we have demonstrated that the induction of miR-34b significantly decreases cell proliferation rate *in vitro *and greatly lowers tumor growth in an orthotopic breast cancer model *in vivo*. We have further demonstrated that the activation of ER by E2 impairs the binding of p53 to the two p53RE regions of the miR-34b promoter leads to repression of miR-34b expression.

In this study, we observed that the expression of miR-34b was downregulated by ER signaling, thus we provide evidence of the negative correlation between ER and miR-34b expression levels in patients with ER+ tumors (Figure 1B). The clinical data derived from our experiments show no significant correlation of miR-34b in ER- tumors (Figure 1C). We believe that miR-34b is not likely the only factor involved in tumor progression in ER- breast cancer patients. Since the malignancy in ER- breast cancer patients, and even in patients with triple-negative breast cancer, is quite complicated and since multiple factors might be involved, the malignancy does not rely only on miRNAs. Researchers in recent studies have pointed out that numerous signaling pathways, including the epidermal growth factor receptor, HER2, IGFR and BRCA1/2 pathways [3,36,37], could contribute to the malignancy of ER- breast cancer. On the other hand, ER+ breast cancer cells are not as malignant, and, in addition, the patterns of their malignancy are usually simpler than those of ER- cells, which may be the reason why the miR-34b expression pattern in ER+ cancer patients is more consistent and more important than that in ER- cancer patients.

Previous studies indicated that miR-34b/c expression is regulated by p53, which mediates cell-cycle arrest and promotes apoptosis [17,38]. DNA damage or aberrant cell-cycle progression leads to rapid increase of p53, which in turn may lead to p53-dependent miR34b expression [16]. Overexpression of miR34b decreases the expression of a number of cell-cycle regulatory proteins, including cyclin D1, c-MET and CDK4 [15,17,23], and thus hampers cell-cycle progression [15,16]. In this study, we confirmed that p53 participates in miR-34b regulation and leads to cell growth inhibition by targeting cyclin D1. In addition, our computational analysis of miR-34b/c targets using PicTar and miRanda pointed to the Notch ligand JAG1 as another possible target. In agreement with these data, we found that miR-34b inhibited breast cancer cell proliferation by targeting cyclin D1 and JAG1.

Although many reports have described the regulation of the miR-34 family as being mediated mostly through the p53 signaling pathway [15-17,39], miR-34b/c could also be regulated by DNA methylation of CpG islands [18,40,41]. Our results show that treatment with 5-aza-dC (a DNA methyltransferase inhibitor) did increase the expression of miR-34b/c, but that the methylation status of miR-34b/c promoter was not affected by E2 treatment. Thus it seems unlikely that E2 regulates miR-34b/c expression through epigenetic regulation. Given that E2 significantly repressed the binding of both p53 and ER to the p53 binding sites in the miR-34b/c promoter, E2's binding to ER may change the conformational structure of ER, which in turn alters p53's binding to the miR34-b/c promoter because of ER-p53 protein-protein interactions.

TAM has been used to treat breast cancer for over 20 years [42]. It has been shown that breast cancer patients bearing ERα+ and wild-type p53 phenotypes respond better to TAM therapy [43,44]. In contrast, breast cancer patients bearing p53 mutations have a worse prognosis and tend to develop drug resistance earlier [45]. Since the regulation of miR-34b/c is dependent on both active ERα and p53, it provide more hints that ER+ breast cancer patients with wild-type p53 expression might be more responsive than patients with mutant p53 to TAM therapy. On the other hand, TAM is a well-known mixed antagonist/agonist of ER. Depending on cellular context, TAM may act as an intermediate agonist or antagonist [42]. In the endometrium, TAM serves as an agonist to activate gene transcription and thus contributes to the elevated incidence of endometrial tumors in women [46]. We have examined the regulation of miR-34b in both OVCAR4 and endometrial cells. We found that E2 exerts similar miR-34b repression effects in human ovarian cancer cell OVCAR4 cells and mouse primary cultured endometrial cells, suggesting that a general repression mechanism by miR-34b may exist in different tissue types.

Current clinical management of breast cancer relies on various pathological markers to provide suitable therapy. In our study, miR-34b has been showed to inhibit cell growth by targeting cyclin D1 and JAG1, both of which are crucial genes covering various types of breast cancer [27,47,48]. Moreover, overexpression of miR-34b leads to inhibition of cell growth in breast cancer cells with different ER and p53 status. These results indicate that miR-34b could be a potential therapeutic target in the treatment of various types of breast cancer.

## Conclusions

In summary, E2 may enhance breast cancer growth by modulating cell-cycle-related genes through the repression of miR-34b. These findings suggest that miR-34b can be considered a new marker for the diagnosis of ER+ breast cancer. Furthermore, miR-34b might be used as a therapeutic target for treating breast cancer.

## Abbreviations

α-MEM: minimum essential medium α; BSA: bovine serum albumin; BSP: bisulfate-specific polymerase chain reaction; ChIP: chromatin immunoprecipitation; ER: estrogen receptor; FBS: fetal bovine serum; FCS: fetal calf serum; H & E: hematoxylin and eosin; HER2: human epidermal growth factor receptor 2; mAb: monoclonal antibody; miRNA: microRNA; MSP: methylation-specific polymerase chain reaction; MTT: 3-(4,5-dimethylthiozol-2-yl)-2,5-diphenyltetrazolium bromide; PBS: phosphate-buffered saline; PR: progesterone receptor; RT-PCR: reverse transcriptase polymerase chain reaction; SERM: selective estrogen receptor modulator; snRNA: small nucleolar RNA; UTR: untranslated region.

## Competing interests

The authors declare that they have no competing interests.

## Authors' contributions

YML carried out all the *in vitro*, *in vivo *and clinical studies, participated in performing the reporter assay and drafted the manuscript. JYL participated in the design of the study and approved the manuscript. CCH helped to design, and offered suggestions for, the clinical studies. QSH designed and performed the plasmid construction of the reporter assay. SLY made contributions to the analysis and interpretation of data. CRT made substantial contributions to the conception of the study. PCY contributed intellectual input to the design, implementation and interpretation of the results. HWC conceived the study, participated in its design and coordination, and helped to draft the manuscript. All authors read and approved the final manuscript for publication.

## Supplementary Material

Additional file 1 **Supplementary Table 1 Sequences of primers used in this study**. Additional experimental data and sequences of primers used in cloning and quantitative RT-PCR. Click here for file

Additional file 2 **Supplementary Figure 1 Cell viability of Mock and pTRE-miR-34b/Tet-On in the presence or absence of doxycycline (Dox) (2 μg/ml) were detected by performing a 3-(4,5-dimethylthiozol-2-yl)-2,5-diphenyltetrazolium bromide (MTT) assay (*n *= 3)**. Click here for file
