# Supplementary material for: miRNA-34b as a tumor suppressor in estrogen-dependent growth of breast cancer cells
Source: Breast Cancer Res. 2011 Nov 23;13(6):R116. doi: 10.1186/bcr3059 (PMC3326558; doi:10.1186/bcr3059)
Supplement: Additional file 1 — Supplementary Table 1 Sequences of primers used in this study. Additional experimental data and sequences of primers used in cloning and quantitative RT-PCR. [file bcr3059-S1.DOC]

**Supplementary Table 1. Sequences for primers used in this study**

|  | sense | antisense |
| --- | --- | --- |
| **Methylation specific PCR** |  |  |
| **miR-34b-MSP-U** | TGGTTTAGTTATGTGTGTTGTGT | CAACTACAACTCCCAAACAATCC |
| **miR-34b-MSP-M** | TTTAGTTACGCGTGTTGTGC | ACTACAACTCCCGAACGATC |
|  |  |  |
| **Bisulfite Sequence** |  |  |
| **miR-34b-BSP** | GGTTGGGAATTGAAGTTTG | TCAACTAATACTACCTACAAACC |
|  |  |  |
| **Chromatin IP** |  |  |
| **miR-34b-CHIP-1** | GAATCTGGTGCACTCTTACTAATGA | CAATTCAGTGCCTTTGAAGAACTAA |
| **miR-34b-CHIP-2** | AGGTGCCTTCCTTGTACTGTCTA | GAAACTCCAAGGATCTCATTCCC |
|  |  |  |
| **Promoter assay** |  |  |
| **miR-34b-Promoter** | GCTAGCGTGAGGATAAAGAGAACATTCCTTATCCTCTCCT | AAGCTTTCATCTTCTGTGACTCCTAGAGAGAGCTAACAGT |
|  |  |  |
| **RT-PCR** |  |  |
| **Full length ERα** | GCAGGTGCCCTACTACCTGGAGAAC | TGGAGCGCCAGACGAGACCAATC |
|  |  |  |
| **Tet-on miR-34b plasmid** |  |  |
| **pTRE-miR-34b** | AGGCGTATCACGAGGCCCTTTCGT | TATTACCGCCTTTGAGTGAGCTGA |
|  |  |  |
| **Luciferase assay plasmid** |  |  |
| **Luc-JAG-1-3′UTR-WT** | ACTAGTcagaccgcgggcactgccgccgcta | ACGCGTTGGCCATTAATCCAGTGGTGTTTATTC |
| **Luc-JAG-1-3′UTR-Mut** | gggtataataggctctCGGACacagaggga | ctccatccctctgtGTCCGagagccta |
| **Luc-CyclinD1-3′UTR-WT** | CTCGAGgggcgccaggcaggcgggcgccaccg | ACGCGTTCGGAACCGAACTTAGGTTGAGTACCC |
| **Luc-CyclinD1-3′UTR-Mut** | ttttacaatgtcatataGACGGatgtacta | aactaaaactagtacatCCGTCtatatgaca |
|  |  |  |
