# Supplementary material for: miRNA-34b as a tumor suppressor in estrogen-dependent growth of breast cancer cells
Source: Breast Cancer Res. 2011 Nov 23;13(6):R116. doi: 10.1186/bcr3059 (PMC3326558; doi:10.1186/bcr3059)

**Supplementary Figure 1**

Cell viability of Mock and pTRE-miR-34b/ Tet-on in the present or absent of doxycyclin (2 μg/ml) were detected by MTT (n = 3).


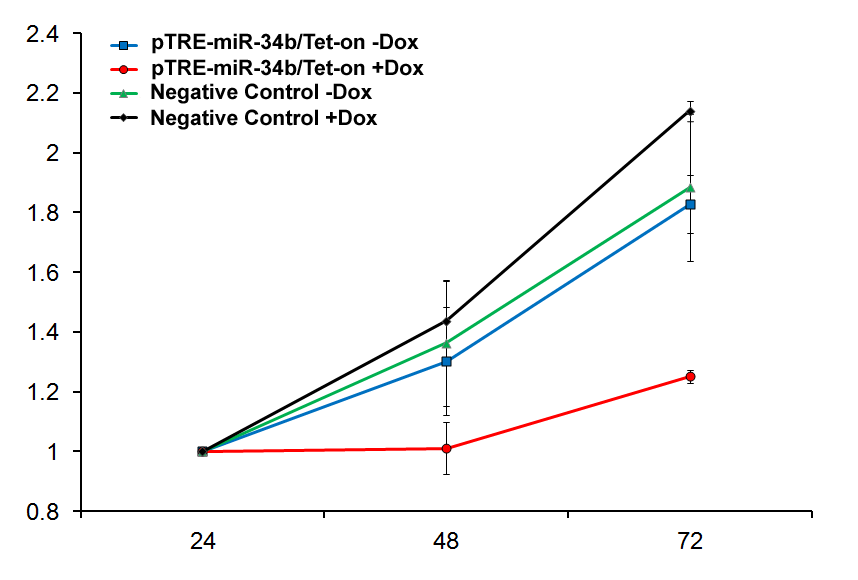

Supplement: Additional file 2 — Supplementary Figure 1 Cell viability of Mock and pTRE-miR-34b/Tet-On in the presence or absence of doxycycline (Dox) (2 μg/ml) were detected by performing a 3-(4,5-dimethylthiozol-2-yl)-2,5-diphenyltetrazolium bromide (MTT) assay (n = 3). [file bcr3059-S2.DOC]
